# Ten simple rules for succeeding as an underrepresented STEM undergraduate

**DOI:** 10.1371/journal.pcbi.1010101

**Published:** 2022-06-09

**Authors:** Melanie Duc Bo Massey, Suchinta Arif, Shanukie Embuldeniya, Karma Nanglu, Joseph Bielawski

**Affiliations:** 1 Department of Biology, Life Sciences Centre, Dalhousie University, Halifax, Canada; 2 Museum of Comparative Zoology and Department of Organismic and Evolutionary Biology, Harvard University, Cambridge, Massachusetts, United States of America; 3 Department of Mathematics and Statistics, Dalhousie University, Halifax, Canada

## Abstract

Undergraduate students from underrepresented backgrounds (e.g., Black, Indigenous, and people of color [BIPOC], members of the Deaf community, people with disabilities, members of the 2SLGBTQIA+ community, from low-income backgrounds, or underrepresented genders) continue to face exclusion and marginalization in higher education. In this piece, authored and edited by a diverse group of Science, Technology, Engineering, and Mathematics (STEM) scholars, we present 10 simple rules for succeeding as an underrepresented STEM undergraduate student, illuminating the “hidden curriculum” of STEM specifically as it relates to the underrepresented undergraduate experience. Our rules begin by encouraging students to embrace their own distinct identities and scientific voices and explain how students can overcome challenges unique to underrepresented students throughout their undergraduate degrees. These rules are derived from a combination of our own experiences navigating our undergraduate STEM degrees and the growing body of literature on improving success for underrepresented students.

## Introduction

If you are Black, Indigenous, and a person of color (BIPOC), a member of the Deaf community, someone with a disability, a member of the two-spirit, lesbian, gay, bisexual, trans, queer, questioning, intersex, and asexual (2SLGBTQAI+) community, from a low-income and/or first-generation background, or a woman, femme, nonbinary, or genderqueer individual, you are likely underrepresented in the fields of Science, Technology, Engineering, and Mathematics (STEM). Due to historic and contemporary exclusion and marginalization, underrepresented groups continue to face significant challenges in STEM [1–4]. Among these challenges are damaging stereotypes, hostile educational environments, discrimination, and complexities related to socioeconomic status [5–7]. Nonetheless, underrepresented scholars continue to lead the charge in pushing for equity, diversity, inclusion, and the exchange of diverse ideas within scientific disciplines [8,9].

Underrepresented scholars bring myriad benefits to STEM and scientific culture. Scholars from diverse backgrounds have made countless meaningful contributions to science, increasing the novelty, impact, and quantity of scientific discoveries [10–12]. Furthermore, increasing the diversity of identities in STEM has positive implications for the well-being of existing diverse scholars, contributing to higher resilience, motivation, and confidence in their careers [13].

Despite the benefits underrepresented students bring to STEM, there continue to be systemic and institutional barriers that negatively impact their success and retention in science education. This is particularly true at the undergraduate level. The aim of this piece is thus to provide advice that can aid underrepresented undergraduate students in successfully navigating their STEM degrees, paying particular attention to the systemic barriers they may face.

In addition, our rules make explicit the “hidden curriculum” [14] of science. These are the unwritten rules and norms that students are expected to conform to, which can perplex those who are new to the field but are easily navigated by dominant groups. Whether or not students are familiar with these systems can have important ramifications for long-term success and feelings of belonging in academia [15]. For example, knowledge of the hidden curriculum can influence students’ comfort in professional networking scenarios, their ability to develop a support network, their knowledge of how to apply for scholarships and work opportunities, and the educational outcomes of their degrees [15]. For underrepresented scholars, this hidden curriculum is intertwined with systems of prejudice and exclusion that unfortunately still exist within academia. We therefore provide advice that acknowledges this unique intersection of challenges faced by underrepresented students.

This piece is authored and edited by a group of diverse STEM graduates. Among us are scholars who are racialized, first-generation immigrants, queer, working class, have experienced mental illness, are women and femmes, or a combination of these identities. We have each faced—and continue to face—challenges on our paths in academia. Here, given our collective decades of experience navigating academic institutions, we have consolidated essential advice for underrepresented undergraduates to persist and succeed in STEM.

### Rule 1: Own your identity

Coming from an underrepresented background, you may experience significant inequitable challenges during your undergraduate degree. In the face of these challenges, it will be important to remember that your identity as an underrepresented student does not diminish your worth: Instead, it is an asset. Your identity and lived experiences add intrinsic value to your perspectives. And, as a postsecondary STEM scholar, you have an exciting opportunity to learn the language of science, imparting you with tools that will help you use your valuable scientific voice to influence science and society.

As you continue your journey through higher education, there may be times when you need validation and strength. There are a few mantras you can remind yourself of during these worrisome periods. First, “I am not an impostor” [16]: You belong here, and you earned your position here. Next, “My identity is powerful”: You are not required to assimilate into the majority to be successful [8,9]. Importantly, “I am valuable”: Your unique perspective can bring immeasurable benefits to science [10].

### Rule 2: Create a long-term plan guided by a vision

Your “scientific vision” is a guide that will help you attain your goals in STEM. Your vision is driven by your personal ambitions, as well as your unique identity and lived experience (e.g., family and community values, heritage, and role models). Some of you will have specific scientific goals, prosocial social motivations, or combinations thereof. You may envision many roles for yourself in society. It is unimportant whether your vision aligns with the stereotypical definition of who a scientist is—the unique combination of goals and ambitions that shape your vision are intrinsically valuable and useful to society [17]. Whatever your vision may be, the key is to transition your vision into a concrete plan for the successful completion of your undergraduate science degree and to lay out foundations for your future goals.

If you’ve never thought about your vision, you may want to ask yourself the following introspective questions:

1. What aspects of science am I most passionate about? (e.g., discovery, technology development, education, outreach, policy, or ethics)
2. What do I enjoy learning about the most? (e.g., the basic sciences, the applied sciences, mathematics and statistics, computation and analysis, or science history and culture)
3. What impact do I want to have on nature, technology, as well as local and global communities?

These questions can help you identify elements of STEM that are interesting and fulfilling to you. Upon reflection, you may decide that your vision is to pursue fields such as graduate studies, industry positions, medical school, or teaching. You may instead have a broader vision, perhaps involving overarching ideas like helping your community or tackling global issues (e.g., wildlife conservation). Your vision at this time may be as broad or narrow as you feel is right, and it will evolve as you and your view of the future change.

Once your current vision is outlined, the next step is to develop a plan for your undergraduate degree, guided by your vision. Again, we suggest you start by asking yourself a few simple questions.

1. What are my goals, both in the short term and long term?
2. What new skills or behaviors will help me to achieve my goals?
3. How, where, and when do my goals align with my academic program, and how can I leverage my academic and cocurricular programs to help me achieve these goals?

At this stage, it’s likely that you will have only a few goals, and it is natural to be a little uncertain about what to do next. In this case, you could consider making an appointment to see an academic advisor or mentor early in your degree, to help create an effective plan for your undergraduate degree. They will be an invaluable resource for discovering skills, courses, and experiences that are both relevant and personally enjoyable [18]. Share your vision and goals with a trusted academic advisor or mentor and ask for their help identifying activities that will facilitate achieving your goals (but also see Rule 8).

**Fig 1:**
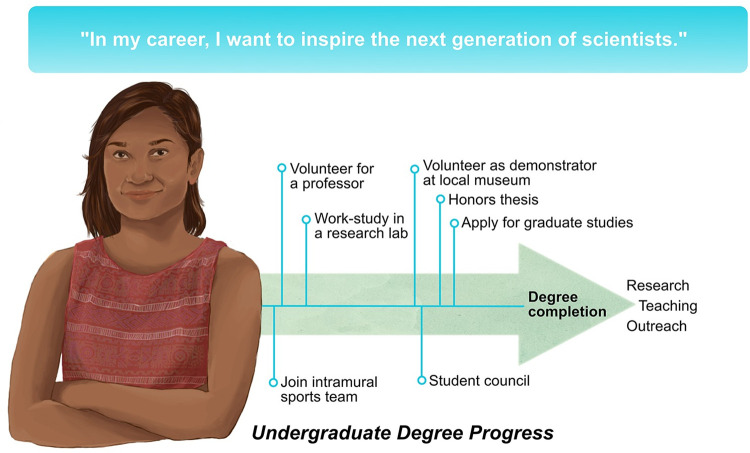
An example of a “vision,” which a student might start to develop as they enter their undergraduate STEM program. A vision statement need not be specific: This student, for example, knows that they want a career in which they can “inspire the next generation of scientists,” which can include research, teaching, and outreach. The plan details the important goals and milestones that will provide valuable experience in these domains and arranges these goals such that there is a variety of experiences, and that experiences can build upon one another. The goals are arranged in a relative timeline that tracks their undergraduate degree completion. The student’s plan also makes space for important social and leadership opportunities. As the student develops, they can refer back to their plan and alter it accordingly. STEM, Science, Technology, Engineering, and Mathematics.

Ultimately, you want to write out your brief plan (a short outline is usually enough) and develop a timeline of goals (e.g., Fig 1) that will guide you through your undergraduate degree. Among the goals on your timeline, you may want to include essentials related to your degree (e.g., course requirements), as well as one or more noncompulsory activities (e.g., clinical volunteering, a semester of work–study, and faculty-supervised undergraduate research). It will also be important to allocate time for nonacademic interests and lifestyle in your planning, prioritizing physical and mental health, as well as keeping in touch with family and friends. For example, a student whose vision involves veterinary science may want to allot time toward doing well in essential courses, working at an animal hospital for experience and financial compensation, and joining a university wildlife club. A holistic approach to planning will ensure that you achieve your vision while making your student years some of the best of your life.

Because your plan is a work in progress, it does not have to be comprehensive. It is important, however, to periodically reassess and update your plan as you hone or even change your interests. Over time, you will expand your circle of friends, mentors, and academic advisors: You should regularly inform them of your goals and seek their advice. Keeping your plan updated will help you stay proactive about deadlines and time management, and it will also allow you to take advantage of new opportunities while keeping on track with requirements for graduation.

### Rule 3: Be critical of your environment

Science culture often prides itself on being inherently objective and neutral. However, research has shown that STEM fields continue to favor Eurocentric and male perspectives, while undervaluing other scientific knowledge systems [19,20]. Similarly, scientists, professors, and people within higher education can operate with intrinsic biases and compromised ethical reasoning. For example, there are countless examples of underrepresented students being met with microaggressions and discriminatory actions by professors both in classrooms and within research labs [9,21]. An important lesson many of us have learned over many decades in academia is that authority figures are not infallible, and that, especially as underrepresented scholars, it is integral to exercise critical thinking and judgment to assess whether our own interests are being prioritized under the institutional and academic hierarchies.

Learning to develop acute reasoning and critical thinking abilities early in your academic career will help you discern what is truly beneficial for your growth as a student and what is not. In addition, critical thinking is an important element of the day-to-day of working scientists. Be mindful of what you are asked to do, ensuring that expectations are reasonable and benefit your goals. For example, you may want to set boundaries on your extracurricular projects or work that allow you ample time for prioritizing your coursework. It is also important to be critical of what you are being taught in classes or by authority figures and reassess questionable material or interpretations; structural prejudice sometimes works to reinforce myths about neutrality and equal opportunity in science [22]. Make an effort to understand the structures and hierarchies of academia, because this understanding can facilitate the voicing of your opinions and concerns.

Ultimately, and with ongoing practice, you should aim to align yourself with mentors who have your best interests at heart and allow you to flex your scientific lens. Some universal “green flags” for both authority figures and colleagues may include: openness to criticism, a pattern of respect, demonstrable empathy, and interest in the success of their students and others. In contrast, “red flags” can include weak work–life boundaries, prejudicial or unkind actions and words, favoritism of certain students, intense micromanaging, unwarranted application of guilt (e.g., unreasonable overtime), or a high degree of nonresponsiveness (e.g., ignoring important emails). Stressful environments can be avoided by learning to recognize early on those traits that conflict with your personal work style and values. When in doubt, talk to those who work closely with these authority figures (other undergraduate or graduate students) to determine if their personalities and mentoring styles would be a good fit for you.

### Rule 4: Demand dignity and self-advocate

There also may be times when individuals or systems within your undergraduate institution will behave in unsupportive, prejudiced, or discriminatory ways toward you or other underrepresented students. It is an unfortunate fact that the systems of power promoting these behaviors exist, but you must learn to navigate them, and regularly remind yourself that you deserve respect and dignity. Historically, underrepresented scholars have self-advocated with great wariness due to the negative repercussions of speaking up against discrimination (e.g., [23]). However, many academics now encourage underrepresented scholars to “speak up every time” [8], and we are cautiously optimistic that academic society is moving toward better handling of prejudice and discrimination.

In situations where others are exercising discrimination against you or your peers, you should not feel guilty about defending yourself or being an ally. You may find yourself wanting to discuss the incident with trusted mentors, allies, or your circle of support (see Rule 8). Among these people, you will find that open communication with those who hold more institutional and systemic power (graduate students, faculty, and deans) and individuals with shared experiences will be immensely valuable [24,25]. However, we recognize that, especially in this early stage of your career, you may not feel comfortable speaking up under the established structures of your institution. In these situations, you could choose to document the incident(s) for potential future use, but, ultimately, we encourage you to protect yourself going forward by seeking appropriate resources as needed (e.g., university counseling services and having a strong peer support group) and continuing to establish your community of allies.

You may also need to self-advocate for necessities related to other educational barriers you may face. These could include, but are not limited to, homework or writing assistance, academic accommodations, extensions on assignments, counseling, financial aid, or tools (e.g., software or technology). To start, acquaint yourself with the structure of your institution as it relates to those needs: Learn who the appropriate contacts are (e.g., Disability Office, Writing Centre, or Financial Aid Office), and open lines of communication with them about your anticipated needs. Be aware of deadlines (e.g., for financial aid). Try to learn what technological resources are available to you: The library is a great place to start, and librarians are very helpful for navigating print and technology resources. To maximize help from your teachers, do as much of the work on your own or with peers as possible, so that you are able to explain to your teaching assistant or professor what you already tried if you still have questions. Indeed, peer tutoring has been shown to benefit student performance in undergraduate courses [26]. Do not be ashamed to ask for extensions: Ask the appropriate contact well in advance, and be polite and brief—perhaps giving a short reason (e.g., “my part-time job required extra shifts this week”). Overall, your institution should have many resources available to help you (see Rule 7), and learning to navigate them early will benefit you significantly in the long run.

### Rule 5: Be efficient with your time and effort

Within the scientific community, scholars from underrepresented backgrounds can often be held to higher standards than majority scholars [23]. We do not mean to suggest that these unequal expectations are just or that you should have to work harder than a majority student to meet them. However, it is important to know that there may be people or systems in academia who harbor these views, and you can prepare yourself to succeed in spite of them.

We encourage you to start by reframing expectations to be your own: Recall that you are in command of setting and realizing your own standards, for your own intents and purposes. Continue to refer to your planned vision and stick to the clear and achievable goals and milestones within that vision. These goals are ultimately the ones that will bring you the strongest career-enhancing benefits and thus maximize the efficiency of your work.

Work in ways that leverage your strengths: Developing good study habits and time management will be crucial. This could mean finding systems that work for you (examples to look into include: the Cornell note-taking system, the Pomodoro method, flashcards, agenda keeping, or study groups) and using those systems to make your work time more efficient. As you proceed in your undergraduate program, it will be important to consistently evaluate your strategies and be cognizant of what works well for you [27,28].

Another key lesson that many of us have learned during our time in academia is the importance of saying “no.” Because of the sheer number of options available to you in your degree, you will be met with many exciting activities that harness your passions, your strengths, or both. When met with these opportunities, ask yourself whether they align with your vision, if they will further your career, entertainment, or social agendas, and what benefits they will bring you. Don’t be afraid to turn down optional work that will add too much to your plate. As an underrepresented student, you may be asked to participate in “hidden labor” [29]. Hidden labor is additional work that is intrinsically important and beneficial to society, but may not be immediately helpful for your own career, financial stability, or degree progress. For example, you may be asked to bake cookies for your department’s fundraiser, do behind-the-scenes work at a volunteer organization (e.g., cleaning), or supervise children at a STEM outreach activity. Although this work is valuable, it can be time-consuming “busy work.” Ultimately, it may not impart significant or relevant experience to you, instead representing an additional burden of labor. Therefore, we recommend selectivity that balances the benefits to both society and your career. Generally, positions that come with a title, another form of recognition, and financial compensation should be prioritized, because they will bring more benefits to you when applying for jobs, internships, scholarships, or other programs.

### Rule 6: Elevate your career while meeting financial needs

As an underrepresented student, you may find yourself having to work throughout your studies to financially support yourself [30]. Acquiring a paying position that also contributes to your academic resume allows you to meet your vision while also attending to your financial needs. There are various options for funding that you can take advantage of during your undergraduate program and choosing them will depend on what is best for meeting your vision, keeping in mind what your current experience level is amenable toward (Table 1).

**Table 1:** Examples of different types of funding that serve the dual purposes of elevating undergraduate students’ careers and providing for their financial needs.

Type of funding	Definition and examples	Benefits
Awards and research scholarships	Funding for students that generally require a competitive application. May fund summer research projects (see below) or be awarded without any work requirements. Examples include NSERC USRA, NSF REU, and SURP	Can have a “snowballing” effect, increasing your chances of winning more scholarships. Excellent for visions that include attending a postgraduate program (e.g., graduate school or medical school) and continuing research; note that many require a minimum GPA or other academic achievements
Summer research or field assistantships	Often involve full-time summer work under a faculty supervisor. May be funded by a scholarship (see above) or by a supervisor. May involve literature research, computation, or assisting with lab/field work (e.g., outdoor experimental setups and data collection)	These positions can serve as a springboard for future undergraduate research (e.g., Honors program) and may give you the option of being a coauthor on a publication. Excellent for visions that include attending a postgraduate program and/or research. Good for visions that require technical experience in a particular domain (e.g., industry)
Co-ops, summer positions, and internships	Hands-on experience in a particular field, which may involve a partnership between your university and a government agency or industry connection, or a student job in a STEM organization	Great for developing relationships with mentors and networking with colleagues. Excellent for visions including industry and government work, which will prioritize soft skills (communication and teamwork) as well as certain unique skills or equipment use (e.g., telemetry)
Work–study programs	Typically on-campus, part-time jobs that are reserved for low-income students. They are generally hosted by a faculty supervisor and involve working in their lab (e.g., maintaining *Drosophila* stocks for a genetics lab). They can act as a good entry point for lab/research experience and networking opportunities, but may also involve menial tasks	Work study programs will vary in which skills they allow you to develop depending on placement, so we recommend that you apply for roles that align with your interests and vision. Generally, you should strive to build early relationships with faculty mentors and learn about the inner workings of their research groups. Work–study programs may be especially important as entry points into research-based visions. They are also flexible part-time jobs that blend well with undergraduate schedules
Teaching assistantships and marking positions	Working under a faculty supervisor to assist with running an undergraduate course. Generally reserved for more senior undergraduates who have been successful in the course. Both positions usually involve marking student assessments and monitoring examinations, but TAships can require demonstration (e.g., of lab techniques)	Generally, these positions pay well relative to other part-time student jobs. Good for understanding the foundations of university curricula and assessment design. They impart soft skills (communication, writing, and leadership) that are generally applicable to all visions. Teaching experience is valuable for those who have visions of staying within the academy (graduate school, research, and pedagogy)

GPA, grade point average; NSERC USRA, Natural Sciences and Engineering Research Council of Canada Undergraduate Student Research Awards; NSF REU, National Science Foundation Research Experiences for Undergraduates; STEM, Science, Technology, Engineering, and Mathematics; SURP, Summer Undergraduate Research Program.

Such dual-purpose funding may be competitive due to the significant benefits it can generate and compound throughout your career (e.g., research awards can have “snowballing” effects, enhancing your ability to win future awards). For this reason, it is very important that you prepare your applications well ahead of deadlines and seek feedback from multiple sources (a trusted mentor, peers, and student writing center). Oftentimes, campus positions involving faculty supervision involve detailed discussions about the nature of the work, and some degree of vetting, prior to submitting an application. This is very common, for example, when applying for external funds to support an undergraduate research project. If you have a mentor in mind already, you should contact them about doing a project under their supervision well before when you want to start, and have them coach you through the application process, if one is needed.

### Rule 7: Take advantage of opportunities

Throughout your degree, you will find yourself in a wonderful position to take advantage of noncompulsory activities such as writing workshops, homework tutorials, journal clubs, your department’s seminars, and scientific research itself. Generally, your institution will send out newsletters about events like these (and you should check these regularly), but you can also reach out to your department to ask about seminars and join student societies and clubs that fall within your domain and your interests. In our own undergraduate programs, these myriad opportunities educated us, built our skills, allowed us to grow our social circles, and, ultimately, were a source of fulfillment and happiness. Within reason, try to take advantage of these opportunities, especially those that “don’t feel like work” and have skill- or career-building advantages. In addition to fostering friendships and connections, these opportunities for skill building and learning can help you improve your educational outcomes and dramatically increase the growing body of scientific knowledge you are developing [30,31]. Moreover, your active participation in these opportunities will expose you to a pool of potential peers and mentors who can help you going forward.

### Research opportunities

One of the most rewarding and important experiences you can have as a STEM undergraduate is active participation in a scientific research group. These positions are highly sought after and carry importance within academia. But, more importantly, they are excellent learning experiences [31] that train you in skills like writing, technical laboratory or fieldwork, and importantly, communicating with professors and colleagues. They are especially important for future research careers (e.g., graduate school), but are also beneficial for many STEM careers. Notably, participation in STEM research has been shown to enhance the persistence of minority students in their undergraduate degrees [6].

It’s important to try to acquire research experience early, perhaps by taking a paid position (e.g., work–study) doing directed tasks to start. Begin your search by identifying professors whose research programs are interesting to you, by learning more about their research through their websites and, importantly, by contacting their current students to get a feel for their personalities and expectations. One question you could ask current students is whether the professor has demonstrated a commitment to their students’ success and well-being. If the professor seems like a good fit, reach out politely in an email expressing why you are interested in their lab and what you would like to do (e.g., volunteering in animal care, data processing, or even just sitting in on lab meetings). Have a resume ready. Make sure to meet with them to get a feel for their lab’s culture, and ensure that their expectations are clear and achievable.

As you develop your confidence and hone in on what interests you, you can begin to have more autonomy over the research, perhaps in the form of an independent research course for which you will receive credit or on an undergraduate research scholarship or work program. In addition to credit or compensation for research, you can receive an invaluable letter of recommendation from your supervisor or even find yourself in a position to publish this research (a gold standard that will immensely benefit your career and scholarship options). Research opportunities also generally come with the added bonus of having a small lab network of undergraduates and graduate students, who oftentimes have “lab meetings” where literature and research progress are discussed. This is where the culture of the lab group is important, as its members can also serve as a valuable social support system.

### Skill-building opportunities

There are many skills that scientists need that are not explicitly taught in STEM undergraduate courses, but remain integral to STEM careers and, more broadly, your professional development. Here are several key skills and the opportunities that facilitate the development of these skills:

Written communication. There will be many opportunities to develop your writing skills, including writing workshops (e.g., for scholarship writing) and student writing centers. You can also offer to peer-review your friends’ work and have them review yours, building on each other’s ideas of what works. Most importantly, practice writing whenever you can [32], especially if it reaps additional benefits: For example, consider submitting short pieces on recent science to student newspapers and essay contests and add these to your resume and scientific portfolio.

Oral communication. Senior scientists are often called upon to give talks on their research to the scientific community, local community, and media. Take advantage of opportunities to practice your public speaking in low-stakes environments, like student journal clubs (where you might present a paper you read to other students) and student conferences (where you might talk about research you participated in). You might want to start by giving a “poster talk,” where you create and present a scientific poster to individuals, rather than to a large conference group. Many presentation-style activities also serve the dual purpose of providing helpful feedback on your work.

Scientific literacy. The ability to read and interpret scientific content is extremely important in science and will benefit your studies and career in STEM as well as your scientific literacy in society. You can take advantage of resources that help improve your scientific literacy, such as student journal clubs, which will give you an opportunity to practice reading and even criticizing scientific literature. Attending seminars and conferences, in which speakers will verbally explain research, is also an excellent way to improve your understanding of how science works, while also broadening the scope of your scientific knowledge.

Technical expertise. There are numerous technical skills that scientists become proficient at, depending on their fields. Learning some of these skills can be an asset to your career. These may include programming or statistics (e.g., in R software), lab techniques (e.g., DNA extraction), field techniques (e.g., electrofishing), or even participation in competitions (e.g., robotics). Depending on what STEM interests you have, attending free skill-building workshops provide experience that can build your confidence, skillset, and make your curriculum vitae (CV; a long and detailed resume) more competitive.

### Academic community opportunities

There are also numerous opportunities for you to take part in academic peer communities during your degree. You could, for example, join your department’s student council, giving you excellent opportunities to meet your peers, enjoy social events, develop leadership, and gain experience liaising with administrations. You could also assist with organizing student events, such as student conferences, which provide broadly applicable event management and networking experience. Besides being valuable for your CV, these community-based opportunities can strengthen your sense of belonging within your program and thus your enjoyment and resilience [33].

### Keeping track of your accomplishments

It is very important to keep ongoing documentation of these activities, no matter how minor they may seem. Each opportunity you engage in should be documented and dated with a short description. Many of us keep a “long CV” or portfolio in which we document everything and draw from it to create shorter and more focused CVs when applying for specific roles and scholarships. This method will save you significant time going forward. As you develop mentor and peer circles, you should also continuously solicit feedback on your growing CV, in addition to periodically reviewing your CV to see how it relates to your plan for your vision.

### Rule 8: Anchor yourself in a supportive community

As an underrepresented student, you may find yourself in situations where your voice, identity, and lived experience are not reflected in your academic environment, and, likewise, your experience within an academic environment may not be easily understood by your friends and family back home. From our own experience, academic environments can consequently feel very isolating. It is therefore imperative that you build a community of individuals on or around your campus who share and support your unique experience. You may find it especially helpful to connect with those from a shared identity who are also in your institution, regardless of whether you are working toward the same degree. To get started, you could consider joining inclusive clubs and organizations (e.g., 2SLGBTQIA+, Black student associations, or intersectional book clubs) or connecting with diverse peers and allies (e.g., through hobbyist groups or social circles).

Anchoring yourself in your community will bring a myriad of benefits, including creating safe spaces for you to share your personal experience and having people who understand your struggles navigating your degree first hand [5,25,34]. For example, as an international student, you may benefit from relating to others about financial, social, and academic obstacles that are unique to the international student experience. In addition, your community may be able to provide you with specialized opportunities, such as connecting you to diverse mentors, funding, and academic projects [35].

As you begin to develop your own scientific community around you, you will benefit significantly from mentorship. This mentorship is exceptionally important because academics tend to select for qualities they see in themselves, sometimes to the exclusion of historically underrepresented groups; good mentors will thus help you navigate the biases that exist in the academy. Seek out supportive professors to work with early in your program, as positive student–professor relationships are associated with undergraduate persistence, satisfaction, and completion [31,36].

If you are planning to join a research lab, it is important to note that professors and their associated research labs are social communities unto themselves. By joining a research lab you will also become a part of another community. Selecting the right research lab can thus create a community that will provide you with intellectual and emotional support, teach you how to conduct science, and provide advice on how to become a successful scientist. Speaking to past and current students, especially those coming from underrepresented backgrounds, may help you determine the quality of potential academic mentors. Holistically, it is also important to choose professors who will mesh well with your working style, personality, and goals. We advise that it is the quality and not quantity of mentors that is important; carefully selecting the right professor(s) to work with has the potential to greatly enhance and elevate your career.

### Rule 9: Bring your values to the table

During your undergraduate degree, you may feel that your science education is not always aligned with your core values. Underrepresented students are often motivated by altruistic values, such as connecting with or giving back to one’s community [17,37]. In contrast, the dominant culture of science can be individualistic, which may feel unfulfilling or isolating for underrepresented students in particular [9,38]. In these times, it is important to remind yourself of your long-term vision, how your community can benefit from your science degree, and how science, in turn, can benefit from your values. For example, you may have entered science to become a pediatrician, and give back to a community that needs healthcare professionals. Or you may have entered science to become a conservation biologist, to one day have a leading role in solving the world’s climate and biodiversity crises. Ultimately, a science degree can elevate you to a position of influence, where you can eventually give back to your community and broader society. Keeping your long-term vision in mind can remind you that your voice is important.

It is also important to stay true to your personal values during your science degree, so that you can have a fulfilling undergraduate experience. This can be done by carefully choosing courses that align with your interests and passions, such as taking a student-centered course that allows you to focus on your own scientific questions [39] or an immersive field course that explores a topic you are passionate about. Another way to bring your values to the table is to participate in community-oriented and value-driven student clubs and organizations [40]. Here, you may even choose to take on a leadership role, increasing your sense of connection and impact.

Importantly, you should also seek out professors whose research interests and prosocial goals align with yours. Ultimately, they may be able to provide you with fulfilling opportunities during your degree, including funded research or work positions. For example, as an Indigenous student, you may feel isolated due to the dominance of Western scientific paradigms in academic spaces [1]. Here, you may benefit from connecting with a professor who implements or is a proponent of Two-Eyed Seeing, a framework that integrates both Western and Indigenous scientific paradigms [19,41]. In general, staying connected with peers, professors, and academic organizations who have similar or complementary passions to yours will help you remain fulfilled during your science degree.

### Rule 10: Find balance, overcome failures, and embrace growth

As you continue to progress in your path to becoming a scientist, it is important to enjoy the holistic journey. This period of your life need not, and should not, revolve solely around being a student. Prioritize your health, both physical and mental, and do not sacrifice self-care [42]. Resting, socializing, exercising, eating nutritiously, enjoying entertainment, and having your medical needs met are all important components of your journey.

Down this road, you will face obstacles and even failures. We are no strangers to failure and must remind you that a failure does not put an end to your journey. As members of historically underrepresented groups, it is also useful to note how negative experiences in your STEM education may differentially affect you. Events which may be minor to others may impact you more severely when part of a pattern of repeated stressors related to inequity in STEM [43,44]; some people may to tell you to casually shrug off negative experiences, without consideration of the broader context in which they occurred. These factors are an additional barrier to success for historically underrepresented students, representing an imposed loss in productivity which is often taken to indicate lesser merit.

With that said, during these times, remember that failures are a critically important but often underdiscussed hallmark of the scientific process [45]. Leverage your failures as a tool to devise new and creative ways to overcome challenges [46], acknowledging that they are a natural part of learning [47]. Indeed, those selfsame experiences provide you with unique insights in how to endure in systems hostile to your success. This idea has been described as “navigational capital” [48], representing the significant value you bring to a work force as an advocate who can advise those that follow in your footsteps. Be kind to yourself if you fail, because everyone fails, and failure is an opportunity for growth.

## Concluding thoughts

While this guide is meant to help you succeed in your journey, learning these rules and benefiting from them are not the same thing as blending in. It is our hope that this piece illuminates the “hidden curriculum” of academia, while reinforcing the strengths inherent in your identity. Ultimately, your unique perspective has intrinsic value, and the scientific community will be lesser for its loss.

## Afterword

This piece was originally developed to be an in-class resource for the emerging “Science Scholars and Leaders” program in the Faculty of Science and the Departments of Biology at Dalhousie University, Canada. The program aims to prepare scholars from underrepresented backgrounds with skills, mentorship, and an intimate peer community that collectively increase their retention and success during their undergraduate STEM degrees. To maximize benefits from this paper, we recommend educators introduce it to students early in their degrees, e.g., the first meeting of their first science class. Students would benefit from an open-ended discussion of the piece, where they share their personal experiences and feedback on the rules and critically judge the applicability of the rules to their own situations. It may also be beneficial to invite students to begin developing their visions for their careers and create simple flowcharts that plan goals and milestones.
